# Introduction to the special collection on family changes and inequality in East Asia

**DOI:** 10.4054/DemRes.2021.44.40

**Published:** 2021-05-11

**Authors:** Hyunjoon Park

**Affiliations:** 1 Department of Sociology, University of Pennsylvania, Philadelphia, USA.

## Abstract

**BACKGROUND:**

East Asian societies have experienced rapid social changes, among which the extraordinary expansion of higher education for both women and men, rising economic inequality, and increasing labor market uncertainty should be particularly relevant to family changes. At the same time, gender inequality and traditional gender norms still prevail and shape family life in the region. The eight articles in this special collection share the common interest of how families in East Asia have evolved against a backdrop of growing economic inequality and persistent gender inequality – among other key forces affecting family life – across a variety of family-related outcomes: from singlehood, marriage intentions, and dating, through fertility, the time use of adolescents and parents with young children, and women’s careers, to intergenerational coresidence and the life satisfaction of older parents.

**RESULTS:**

Our eight studies look at connected but distinctive outcomes related to family life, and collectively demonstrate the relevance of increased labor market uncertainty and the continuing male breadwinner norm to emerging patterns of family behavior in East Asia. They illustrate specific ways in which East Asian families are shaped by the joint forces of economic inequality and gender inequality.

**CONTRIBUTION:**

This volume highlights the complexity and heterogeneity of contemporary East Asian families, inviting family and demography researchers to conduct more studies on under-researched family behaviors such as cohabitation and nonmarital births and to revisit the conventional perception that family ties are strong and family support is readily available in East Asia.

## 1. Background

In recent decades, families in China, Japan, and South Korea (hereafter Korea) have experienced rapid changes in many aspects of family life, challenging the traditional image of East Asian families (Park and Woo 2020; Raymo et al. 2015). Marriage has been delayed overall and certain groups of young adults even forgo marriage, while the marriage knot is becoming much looser. Although the fertility decline began at different times in China, Japan, and Korea, the speed of falling fertility rates has been considerable in all three societies. Meanwhile, an increasing number of people in the region live alone (Yeung and Cheung 2015). However, some aspects of family behavior still remain more or less intact despite the rising tide of demographic change. For instance, parenthood is still closely linked with marriage in most East Asian societies (Raymo et al. 2015).

Broad societal changes have likely affected East Asian families. First of all, the dramatic expansion of higher education among women (and men) in the region has opened up more economic opportunities for women (Hannum et al. 2019). However, the persistent gender inequality and discrimination in the labor marker, the gendered division of labor, and hostile work environments, particularly in Japan and Korea, are still major barriers to women’s continued labor market participation after marriage and childbirth (see Brinton and Oh 2019). The persistent gender norms are particularly striking, given the dramatic expansion of higher education for both men and women. In addition, rising economic inequality and deteriorating labor market conditions (e.g., rising job insecurity and the growth of part-time, irregular, and low-paid jobs, often called nonstandard employment – see Raymo, Uchikoshi, and Yoda (2021) and Lim (2021) in this special collection) in East Asia have broad implications across a wide range of life-course outcomes, including young people’s marriage prospects, married couples’ fertility plans, the decision to end a marriage, and older parents’ decision to live alone (see Raymo et al. 2015).

It is a very daunting task to precisely assess the extent to which changing economic contexts (or any other macro factors) are responsible for family changes, especially across societies. An alternative but less satisfactory and rigorous strategy is to carefully look at how individuals and families at different positions in the socioeconomic hierarchy of a given society show varying patterns of family behavior in the contexts of rising economic inequality and worsening labor market conditions. Moreover, a comparative perspective across East Asian families that share some features (particularly the persistent male-breadwinner norm) and are experiencing similar macro-economic changes may provide additional insights on the issue, even though the economic context certainly differs across East Asian societies.

The following eight articles of this special collection, titled Family Changes and Inequality in East Asia, share a key interest: how growing economic inequality and labor market uncertainty and stubborn gender-inegalitarian norms and environments have shaped families in East Asia. Collectively, these eight articles address how families in East Asia have evolved against a backdrop of changing economic contexts and persistent gender inequality, among other key forces affecting family life, across a variety of family-related outcomes: from singlehood, marriage intentions, and dating (Brinton, Mun, and Hertog 2021; Raymo, Uchikoshi, and Yoda 2021; Yu and Hara 2020), through fertility (Lim 2021), the time use of adolescents and parents with young children (Hertog and Zhou 2021; Park 2021), and women’s careers (He and Wu 2021), to intergenerational coresidence and the life satisfaction of older parents (Chen, Shen, and Ruan 2021). Our collection moves beyond focusing on a single event such as marriage or fertility in a short time window to cover different stages of the life course, from singlehood to older parents’ living arrangements. Looking at closely related family outcomes along with the sequence of the life course, our eight studies offer a full picture of how different family behaviors and events are connected, and the similar or differing effects of societies’ economic and gender contexts across different but interconnected family-related outcomes.

Our collection considers three countries in East Asia – China (Chen et al.; He and Wu), Japan (Brinton et al.; Hertog and Zhou; Ryamo et al.; Yu and Hara), and Korea (Lim; Park). Although more papers are devoted to Japan, juxtaposing studies of the three countries highlights both the similarity and heterogeneity in family behaviors in these three East Asian societies. Scholars often point out that East Asia shares certain Confucian family values and gender norms, albeit to varying degrees (Raymo et al. 2015; Yu and Xie 2021). However, China’s specific experience of marketization and its political structure means that caution is required when comparing family life in China with that in Japan and Korea. Another recent special collection in Demographic Research, Life-Course Decisions of Families in China, is a good companion to our special collection and provides in-depth and comprehensive coverage of changes in Chinese families, especially across “multiple life domains” (Xu et al. 2020).

An evident demographic trend in East Asia is delayed and forgone marriage. Among Japanese and Korean men the mean age at first marriage has increased from 28.4 and 27.8 in 1990 to 31.1 and 32.6 in 2015, respectively, while the corresponding increase during the same period among Japanese and Korean women is from 25.9 and 24.8 to 29.4 and 30.0, respectively (National Institute of Population and Social Security Research 2021; Statistic Korea 2017). Meanwhile, China has seen a somewhat modest but steady increase in age at first marriage, from 24 to 26 among men and from 22.1 to 23.9 among women during the period 1990‒2010 (Raymo et al. 2015). The share of never-married 40- to 44-year-old men and women (the majority of whom will likely remain single) has almost tripled among Japanese men and women, from 11.8% and 5.8% in 1990 to 30% and 19.3% in 2015, respectively (United Nations 2017; see also Brinton, Mun, and Hertog 2021). The corresponding increase in the share of never-married Korean men and women among those aged 40–44 during the same period is even more remarkable: from 1.5% and 1.1% to 22.5% and 11.3%, respectively. The share of singlehood is comparatively low in China: in 2010 it was only 4.2% and 0.8% among 40- to 44-year-old men and women (United Nations 2017).

As the transition to marriage lengthens, an increasing number of young people in East Asia, particularly in China and Japan, are living together before marriage. Traditionally, pre-marital cohabitation has not been socially accepted in the region and therefore there is a dearth of research on this topic. However, the small body of existing research reports a steady increase in pre-marital cohabitation in China and Japan (Yu and Xie 2021; Raymo, Iwasawa, and Bumpass 2009). Meanwhile, in Korea cohabitation before marriage is still socially taboo, despite increasingly open attitudes. In East Asia pre-marital cohabitation tends to result in formal marriage, suggesting that cohabitation has not yet been institutionalized as an alternative to marriage (Raymo, Iwasawa, and Bumpass 2009; Raymo et al. 2015). This should be understood in the context of the strong linkage between marriage and parenthood that still prevails in most East Asian societies.

Scholars have drawn on various theories of marriage to explain the declining marriage rates in East Asia. Among other things, the rising economic inequality and growing labor market uncertainty make it difficult for young adults, especially the less-educated, to afford marriage and parenthood (see Park and Lee 2017 for different theories). The deteriorating economic prospects of young East Asian men in particular can be a strong barrier to marriage and parenthood in a context where the traditional gender division of labor and the male-breadwinner model still dominate expectations of both young men and women (see Brinton, Mun, and Hertog 2021; Park and Woo 2020). Along with the growing concentration of income and wealth at the top of the distribution, the increasing difficulty and extended time it takes to find a stable and high-quality job make it hard for young men, especially those with a low level of education, to meet the expectations of family formation determined by the male-breadwinner model. This combination of a deterioration in young people’s economic conditions and the persistent norm of the gender division of labor may explain the larger decline in marriage rates among low educated Japanese and Korean men than that among their more-educated counterparts (Park and Lee 2017; Fukuda, Raymo, and Yoda 2020). Yu and Xie (2021) stress the comparably low marriage rates among low-educated Chinese men, although no specific evidence is presented to assess trends. The prevailing norm of a gendered division of labor in East Asia also helps understand the traditionally negative (albeit changing: see Fukuda, Raymo, and Yoda 2020) relationship between women’s education and marriage.

## 2. This Special Collection

The rapid decline in marriage has especially important implications in East Asia, as several authors in this special collection highlight. As most childbirths happen within marriage, marriage decline readily results in fertility decline (also see Raymo et al. 2015). Against the backdrop of the recent marriage and fertility decline in Japan, the first three studies in our special collection take a deep look at partner preferences, marriage intentions/desires, and the dating of young Japanese men and women. In order to better understand why young people in East Asia delay or even give up on marriage, it is critical to examine their intentions to marry and actual dating behavior.

Yu and Hara (2020) show that various job characteristics such as low income, low levels of job security and autonomy, and workplaces with constant staff shortages are associated with lower levels of intention to marry among single Japanese men. Interestingly, except for low income, these job characteristics are not associated with single women’s marriage intentions. Instead, single women in a workplace where teamwork is emphasized are more likely to intend to marry. The authors argue that “in workplaces where most work is done collaboratively, single women are more likely [to be] exposed to their older and married coworkers’ potentially more conventional family values,” which is conducive to increased marriage intentions (p.1537). Emphasizing the persistent male-breadwinner norm in Japan, the authors find that economic aspects of job characteristics matter more for Japanese men’s intentions to marry while the social aspect matters more for women’s intentions.

This finding further highlights the implications of young people’s deteriorating labor market conditions for their dimmed marriage prospects. Raymo, Uchikoshi, and Yoda (2021) identify three pathways through which young Japanese people change or maintain their marriage intentions/desires over time and end up with either marriage or singlehood: ‘failure to realize intentions/desires to marry,’ ‘rejection of marriage,’ and ‘unplanned drifting into singlehood.’ Combining the marriage intentions/desires and actual marriage outcomes over a period of eight years from a nationally representative longitudinal survey of young adults, the authors empirically present the prevalence of each pathway. The rising economic uncertainties of low-educated men are a key factor leading them to the life-course pathway captured by the ‘failure to realize intentions/desires to marry.’ The share of the marriage rejection pathway is fairly small for both men and women, less than 5% among those who remain unmarried after 8 years, while the unplanned drifting pathway accounts for the largest share (about 60%) of unmarried men and women.

Brinton, Mun, and Hertog (2021) attempt to identify young Japanese men and women’s preferences for partners’ socioeconomic and demographic characteristics. The marriage process is initiated not only by the mere intention to marry but also by specific preferences for potential partners. Using the unique data of customers who used a marriage agency to date a romantic partner, supplemented by an analysis of the interview data of 30 Japanese men and women aged 25 to 34, the authors highlight persistently gendered patterns of partner preferences in Japan. In terms of absolute men’s and women’s socioeconomic characteristics, more-educated and higher-income men receive more dating offers in a linear fashion (i.e., more education and higher income, more offers). However, women with a graduate degree receive fewer offers than women with a bachelor’s degree, while women with the highest level of income receive the fewest offers. The gendered patterns of partner preference are also evident regarding relative characteristics: Japanese women prefer men who are more educated, earn more, and are older than themselves, while men do not care so much about a partner’s relative education and earnings, but do care about age.

Moving beyond singlehood to married couples, the next three papers in our special collection address how changing economic contexts combined with the persistent gender division of labor in East Asia shape fertility, parental time investment in young children, and parental influence on adolescents’ time use. The influence of continuing gender norms is also observed in the fertility patterns of Korean women. Lim (2021) shows that Korean women’s employment status, particularly standard employment, is negatively associated with the odds of having first and second births, while their husbands’ standard employment is positively associated with women’s fertility. The author interprets this finding as reflecting the challenges Korean women face in combining work and family. Noting the lower odds of having a second birth among women whose husbands are in nonstandard employment compared to their counterparts in standard employment, the author finds confirmation of how deteriorating employment prospects deter couples’ decision to have a second child. The interesting relationship between housing arrangements and fertility in Korea highlights the implications of rising economic inequality and housing insecurity for reduced fertility.

Park (2021) and Hertog and Zhou (2021) provide empirical evidence that parents’ and children’s time use are systematically related to the socioeconomic conditions of Korean and Japanese families. Tracking how parents’ time use to care for children under school age has evolved from 1999 to 2014 based on time diary data, Park (2021) demonstrates growing differences in childcare time between parents with a bachelor’s degree and their counterparts with a high school diploma at most. During this period when economic inequality was on the rise and labor market conditions were increasingly volatile, Korean parents, regardless of educational level, generally increased their time investment in children. This increase in parental time spent on childcare may also reflect escalated competition for children’s educational success, where Korean parents’ involvement in cultivating their children’s cognitive, social, and health development is noticeable even among preschoolers (Oh 2020). However, childcare time has increased more among parents at the top of the educational hierarchy than among their counterparts at the bottom, leading to educational divergence in childcare time. Given that parental time is a key resource for children’s education and well-being, this diverging childcare time between more- and less-educated parents suggests potentially increasing educational and social inequality between children (see McLanahan 2004).

Shifting focus to children’s time use rather than parents’ time investment in children, Hertog and Zhou (2021) assess the association between family socioeconomic conditions, represented by household income and parental education, and adolescents’ daily minutes spent on study (inside and outside of school separately), sleep, and leisure in Japan. Adolescents from higher income households study for more hours after school and spend less time on leisure than their peers from less affluent families. The comparison between adolescents with fathers who have and do not have a bachelor’s degree, and between adolescents with mothers who have and do not have tertiary degrees (junior college/professional or university) show the same pattern of after-school study time and leisure time. Notably, mothers’ education seems to matter more for children’s time use than fathers’ education. Similar to Park (2021) for Korea, the authors allude to the implications of more studying hours among adolescents from families with more economic and cultural capital for socioeconomic inequality in the next generation in Japan.

The last two articles turn to China, which had considerably different economic and gender regimes during its socialist period. However, as He and Wu (2021) describe succinctly, marketization has fundamentally changed Chinese society. As the communist work unit (*danwei*) collapsed, individuals were left to compete fiercely for economic success. Moreover, integration of public and private spheres under the danwei system broke up during marketization. The authors emphasize that along with marketization the traditional gender norm, which once had been suppressed by the socialist gender ideology, has revived, reseparating public and private spheres. As marketization has intensified, the authors find increasing penalties to marriage and parenthood among women. Married women’s lower likelihood of upward mobility and greater likelihood of exiting the labor market, relative to unmarried women, has grown more in the last market reform period (1999‒2008) than in earlier reform periods. Similarly, the penalty of motherhood related to involuntary exit from the labor market has increased in the last reform period. Such trends are not found among men.

East Asian families have long been recognized as having strong ties, often indicated by a high prevalence of coresidence between adult children and their elderly parents and available family support for vulnerable members (Reher 1998). Chen, Shen, and Ruan (2021) question the assumption that adult children co-residing with (or living near) their parents is beneficial for the older parents’ life satisfaction. The authors show that proximity to adult children does not matter for the older parents’ life satisfaction per se. Instead, they highlight the importance of the quality of the parent–child relationship in relation to elderly parents’ life satisfaction. When parents have a poor quality relationship with their adult children, living under the same roof or close by does not make a difference compared to living far away. However, the authors caution readers not to conclude that elderly parents’ living arrangements do not matter for their life satisfaction. Living near their children can still be important for life satisfaction among more vulnerable older parents.

## 3. Future research on East Asian families

Looking at connected but distinctive outcomes related to family life, our eight studies collectively demonstrate the relevance of increased labor market uncertainty and the continuing male-breadwinner norm for emerging patterns of family behavior in East Asia. Men’s socioeconomic characteristics are important factors that Japanese women consider in accepting a date offer, and men with less impressive socioeconomic profiles are less likely to intend or desire to marry (Brinton, Mun, and Hertog 2021; Yu and Hara 2020). In Japan the higher prevalence of the ‘unplanned drifting’ pathway to singlehood among those without university education also illuminates how marriage intentions/desires that can affect the transition to marriage are systematically linked to young adults’ socioeconomic conditions (Raymo, Uchikoshi, and Yoda 2021). In Korea the likelihood of having a child increases with men’s standard employment status and other socioeconomic indicators, while women’s socioeconomic characteristics either do not matter much or their being in standard employment actually lowers the odds of childbirth (Lim 2021). The diverging gap in childcare time between the most- and least-educated mothers and fathers in Korea offers another clear illustration of how economic and gender inequalities combine to shape family life. Despite already investing considerable time in children, over the period characterized by growing economic inequality and uncertainty Korean mothers have increased their childcare time even further (Park 2021). When educational competition for intergenerational social mobility intensifies in an uncertain economic environment, the traditional gender ideology underpinning women’s childcare responsibility can easily be evoked in support of the middle-class strategy of intensive parenting (see Bianchi et al. 2012). Hertog and Zhou (2021)’s finding of the relative importance of Japanese mothers’ education over fathers’ education in children’s study and leisure time is also interpreted in this context. Despite its distinctive political structure, family life in a China where marketization is in full swing is similarly shaped by economic and gender contexts. China’s intensifying market economy has invoked the traditional gender division of labor under which marriage and motherhood penalties to women’s job careers are more evident than in earlier periods of marketization (He and Wu 2021). Although parents living away from their adult children in contemporary China does not necessarily lead to them having lower life satisfaction, it can be particularly harmful for older parents who are more disadvantaged (Chen, Shen, and Ruan 2021).

Although our collection deals with various aspect of family behavior, several important family behaviors are missing. In particular, more research on cohabitation and nonmarital births in East Asia is needed. The increasing prevalence of cohabitation and nonmarital births can be a useful litmus test to evaluate changes in the prevailing traditional gender norm in East Asia. It is critically important to identify factors that would loosen the link between marriage and parenthood in the region. As clearly illustrated in the Korean case where the lack of reliable data hinders scientific research on cohabitation and nonmarital births, greater effort is needed to collect better data.

Another fruitful line of future research would be to focus on intergenerational economic and social exchanges and living arrangements. Recent evidence in Korea, albeit limited, appears to suggest that the traditional perception of strong family ties in Korea (and East Asia) should be revisited. A critical limitation of the existing literature is its insensitivity to potential socioeconomic variation in the availability and strength of family ties. A study of divorced single mothers and their children in Korea shows that single mothers with university education, who are supposedly more advantaged than single mothers without university education, are more likely to live with their own parents (after divorce) than their counterparts without university education (Park, Choi, and Jo 2016). This finding may suggest that family support might actually be more available to those who are relatively advantaged than to those who are in more desperate need. Maybe older parents of no-university-educated divorced mothers with young children are extremely vulnerable themselves and therefore cannot be of any help to their divorced daughters. It is plausible that the different availability and strength of family ties by socioeconomic status may become more evident in macroeconomic contexts of rising economic inequality and labor market uncertainty in East Asia.
